# Deep learning for hepatocellular carcinoma segmentation in MRI: A systematic review of models, performance, and challenges

**DOI:** 10.1097/MD.0000000000047061

**Published:** 2025-12-19

**Authors:** Mohammadreza Elhaie, Abolfazl Koozari, Maryam Arjmandi, Nadia Najafizade

**Affiliations:** a Department of Medical Physics, School of Medicine, Isfahan University of Medical Sciences, Isfahan, Iran; b Department of Medical Physics, School of Medicine, Ahvaz Jundishapur University of Medical Sciences, Ahvaz, Khuzestan, Iran; c Department of Medical Physics, School of Medicine, Iran University of Medical Sciences, Tehran, Iran; d Department of Radiooncology, School of Medicine, Cancer Prevention Research Center, Seyyed Al-Shohada Hospital, Isfahan University of Medical Sciences, Isfahan, Iran.

**Keywords:** carcinoma, computer, deep learning, hepatocellular, image segmentation, liver neoplasms, magnetic resonance imaging, neural networks

## Abstract

**Background:**

Hepatocellular carcinoma (HCC) is a leading cause of cancer-related mortality, necessitating accurate segmentation in magnetic resonance imaging (MRI) for diagnosis and treatment planning. Deep learning (DL) models, particularly convolutional neural networks, have shown promise in automating HCC segmentation, yet challenges like dataset limitations and MRI protocol variability persist. This systematic review evaluates DL models for HCC segmentation in MRI, focusing on model architectures, performance metrics, and implementation challenges.

**Methods:**

Following preferred reporting items for systematic reviews and meta-analyses guidelines, we searched PubMed, Scopus, Web of Science, and Cochrane Library for peer-reviewed studies using DL for HCC segmentation in MRI. Inclusion criteria required quantitative metrics (e.g., dice similarity coefficient [DSC]) and human subjects. Two reviewers conducted screening, data extraction, and quality assessment using quality assessment of diagnostic accuracy studies-2. Narrative synthesis grouped studies by architecture and MRI sequence, analyzing performance and challenges.

**Results:**

Of 2462 records, 13 studies met criteria, predominantly using U-Net-based models (e.g., nnU-Net, UNet++). DSCs ranged from 0.61 to 0.954, with transformers and hybrid models showing adaptability. Clinical applications included diagnosis, treatment planning, and risk assessment. Challenges included small datasets (e.g., 19–602 patients), lesion heterogeneity, and MRI protocol variability, limiting generalizability. High risk of bias in patient selection was noted in 8 studies.

**Conclusion:**

DL models demonstrate robust HCC segmentation performance in MRI, but dataset limitations, lesion variability, and imaging inconsistencies hinder clinical adoption. Multi-center datasets, standardized protocols, and hybrid approaches integrating radiologist input are critical for advancement.

## 1. Introduction

Hepatocellular carcinoma (HCC) is the predominant primary liver malignancy and a major global health burden, accounting for significant cancer-related mortality, particularly in patients with chronic liver diseases such as cirrhosis and viral hepatitis.^[1]^ Accurate segmentation of HCC lesions in medical imaging is essential for effective diagnosis, staging, treatment planning, and monitoring therapeutic outcomes. Magnetic resonance imaging (MRI), valued for its superior soft tissue contrast and absence of ionizing radiation, is a cornerstone modality for HCC characterization, leveraging T1-weighted, T2-weighted, and contrast-enhanced sequences.^[2]^ However, manual segmentation of HCC in MRI is labor-intensive, subject to inter-observer variability, and complicated by lesion heterogeneity, MRI artifacts, and protocol variability. In clinical practice, accurate segmentation of HCC lesions plays a pivotal role in multiple stages of patient management. In radiotherapy, it defines treatment margins; in surgical planning, it guides resection strategies; and during post-treatment monitoring, it supports objective response assessment. As such, segmentation is not merely a technical step, but a clinical enabler of precision medicine in hepatology and oncology. Deep learning (DL), particularly convolutional neural networks (CNNs) like U-Net and its derivatives, has revolutionized medical image segmentation by offering automated, accurate, and efficient solutions.^[3,4]^ For HCC, DL models have demonstrated significant potential in segmenting tumors from MRI scans, promising to streamline clinical workflows and enhance patient care. Nevertheless, challenges such as non-standardized MRI intensities, limited annotated datasets, and poor model generalizability across diverse patient cohorts and imaging vendors hinder widespread adoption.^[5]^ While systematic reviews have explored artificial intelligence in liver imaging, such as general segmentation or HCC diagnosis across multiple modalities,^[6,7]^ none have specifically focused on DL-based HCC segmentation in MRI with a comprehensive analysis of model architectures, performance metrics, and implementation challenges. Among DL architectures, U-Net and its derivatives (e.g., UNet++, ResUNet) are widely used due to their encoder-decoder structure and skip connections that preserve spatial features. More recently, transformer-based architectures have gained traction by capturing long-range dependencies through attention mechanisms, potentially improving performance on heterogeneous lesions. These foundational differences influence how models learn features from MRI data and are explored further in this review. Unlike previous reviews, which often encompass broader AI applications in liver imaging or include multiple imaging modalities (e.g., CT, ultrasound) and tasks (e.g., detection, classification),^[6,7]^ this systematic review uniquely concentrates on DL models for HCC segmentation using MRI exclusively. For instance, a recent review on AI in liver imaging addressed segmentation as one of several tasks, with MRI studies comprising only a subset of the analysis,^[6]^ while another focused on CNNs for HCC identification, with minimal emphasis on segmentation.^[7]^ Compared to existing reviews, this study provides a more focused synthesis. Pomohaci et al (2025) reviewed AI applications in liver imaging broadly, including detection and classification, while MRI segmentation received limited emphasis.^[6]^ Azer in a study done in 2019 focused on liver mass classification using CNNs, without delving into segmentation metrics or MRI-specific challenges.^[7]^ In contrast, our review systematically evaluates DL architectures specific to HCC segmentation in MRI, performance metrics like dice similarity coefficient (DSC), and barriers to clinical adoption, providing a specialized and updated analysis. Similar to the structured comparative method used by Yaman et al (2025) in coronary imaging, our review offers a model-centric synthesis with clinical implications.^[8]^ This systematic review prioritizes a detailed evaluation of DL architectures (e.g., U-Net, ResUNet), quantitative performance metrics (e.g., DSC, intersection over union), and MRI-specific challenges, such as protocol variability and dataset limitations. By synthesizing these elements, this review aims to provide a targeted resource for researchers and clinicians, identify critical research gaps, and propose actionable directions for advancing DL-based HCC segmentation in clinical practice.

## 2. Methods

### 2.1. Study design and protocol

This systematic review was conducted in accordance with the preferred reporting items for systematic reviews and meta-analyses (PRISMA) guidelines to ensure comprehensive and transparent reporting.^[9]^ The primary aim was to evaluate DL models for HCC segmentation in MRI, focusing on model architectures, performance metrics, and implementation challenges. Ethical approval was waived for this systematic review as it did not involve direct research on human subjects or the use of identifiable patient data.

### 2.2. Eligibility criteria

Studies were included if they met the following criteria: utilized DL models, such as CNNs, for HCC segmentation in MRI; reported quantitative performance metrics, such as (DSC), intersection over union (IoU), or sensitivity; involved human subjects with confirmed HCC; were peer-reviewed original research articles published in English; and used MRI as the primary imaging modality, including T1-weighted, T2-weighted, or contrast-enhanced sequences. Exclusion criteria encompassed: studies focusing on other imaging modalities (e.g., CT, ultrasound) or non-segmentation tasks (e.g., detection, classification); non-DL methods (e.g., traditional machine learning, manual segmentation); reviews, editorials, or conference abstracts; studies lacking quantitative segmentation metrics; and non-English publications or studies on non-human subjects in Table 1.

**Table 1:** Eligibility criteria summary table.

Inclusion criteria	Exclusion criteria
DL-based segmentation of HCC in MRI	Non-DL methods (e.g., traditional ML)
Reported quantitative metrics (e.g., DSC)	Other imaging modalities (e.g., CT, US)
Human subjects with confirmed HCC	Reviews, editorials, abstracts
Peer-reviewed original research	Non-English or animal studies

DL = deep learning, DSC = dice similarity coefficient, HCC = hepatocellular carcinoma, MRI = magnetic resonance imaging.

### 2.3. Search strategy

A systematic literature search was performed across PubMed, Scopus, Web of Science, and Cochrane library to identify relevant studies published from January 1, 2015 aligning with the introduction of U-Net, a pivotal DL architecture for medical image segmentation.^[10]^ The search strategy was structured using the population, intervention, comparison, outcome framework to ensure comprehensive coverage, as detailed in Table 2. Search terms combined medical subject headings and free-text terms, linked with Boolean operators (“AND,” “OR”) and wildcards (e.g., “segment*”) to enhance sensitivity. For PubMed, medical subject headings terms such as “carcinoma, hepatocellular,” “MRI,” and “neural networks, computer” were used alongside free-text terms. Scopus and Web of Science searches were optimized with field-specific queries (e.g., TITLE-ABS-KEY in Scopus), while Cochrane library emphasized technical terms like “CNN.” Reference lists of included studies and relevant reviews were manually screened to identify additional studies.

**Table 2:** Search strategy based on PICO framework.

PICO component	Description	Search terms
Population	Patients with confirmed HCC undergoing MRI	(“hepatocellular carcinoma” OR “HCC” OR “liver cancer” OR “primary liver malignancy”) AND (“MRI” OR “magnetic resonance imaging”)
Intervention	DL models for HCC segmentation in MRI	(“deep learning” OR “convolutional neural network” OR “CNN” OR “U-Net” OR “ResUNet” OR “neural network” OR “artificial intelligence”) AND (“segmentation” OR “delineation” OR “tumor segmentation”)
Comparison	Not applicable (no specific comparator required)	Not applicable
Outcome	Quantitative performance metrics and reported challenges	(“Dice similarity coefficient” OR “DSC” OR “Intersection over union” OR “IoU” OR “sensitivity” OR “specificity” OR “accuracy” OR “performance metrics” OR “segmentation performance” OR “challenges” OR “limitations”)

Search terms within each PICO component were combined with “OR,” and components were linked with “AND.” Database-specific syntax was applied, and filters restricted results to English-language, peer-reviewed original research articles. The search was supplemented by manual screening of reference lists.

DL = deep learning, DSC = dice similarity coefficient, HCC = hepatocellular carcinoma, MRI = magnetic resonance imaging, PICO = population, intervention, comparison, outcome.

### 2.4. Study selection

Search results were imported into EndNote X9 for deduplication. Two independent reviewers (A.B. and C.D.) screened titles and abstracts against the eligibility criteria, followed by full-text evaluation of potentially relevant articles. Discrepancies were resolved through discussion or consultation with a third reviewer (E.F.). The selection process was documented using a PRISMA flow diagram to illustrate the number of studies screened, included, and excluded, along with reasons for exclusion.

### 2.5. Data extraction

Data were extracted by 2 reviewers (A.B. and C.D.) using a standardized form developed a priori. Extracted variables included: study characteristics (author, year, country, sample size); MRI characteristics (field strength, sequence type, contrast use); DL model details (architecture, e.g., U-Net, ResUNet; training dataset size; preprocessing techniques); performance metrics (e.g., DSC, IoU, sensitivity, specificity); validation methods (e.g., cross-validation, external validation); and reported challenges (e.g., dataset limitations, MRI protocol variability, generalizability). Discrepancies in data extraction were resolved through consensus or third-party adjudication (E.F.).

### 2.6. Quality assessment

The methodological quality of included studies was assessed using the quality assessment of diagnostic accuracy studies-2 (QUADAS-2) tool, adapted for segmentation studies.^[11]^ QUADAS-2 evaluates risk of bias (RoB) and applicability concerns across 4 domains: patient selection, index test, reference standard, and flow and timing. Two reviewers (A.B. and C.D.) independently applied the tool, with disagreements resolved through discussion or third-party review (E.F.). Studies were not excluded based on quality scores but were analyzed to explore potential biases affecting reported outcomes.

### 2.7. Data synthesis and analysis

Due to anticipated heterogeneity in DL architectures, MRI protocols, and performance metrics, a narrative synthesis was employed rather than a meta-analysis. Studies were grouped by DL architecture (e.g., U-Net-based, attention-based models) and MRI sequence type (e.g., T1-weighted, T2-weighted, contrast-enhanced). Performance metrics were summarized using ranges and medians where applicable, with emphasis on DSC and IoU as primary segmentation outcomes. Challenges were categorized into technical (e.g., model overfitting, limited training data), clinical (e.g., lesion heterogeneity), and imaging-related (e.g., protocol variability, artifacts) domains. Subgroup analyses explored the impact of dataset size, validation strategies, and MRI field strength on model performance. Gaps in the literature, such as underreporting of generalizability or external validation, were highlighted to guide future research.

## 3. Results

### 3.1. Study selection

**Figure 1.**
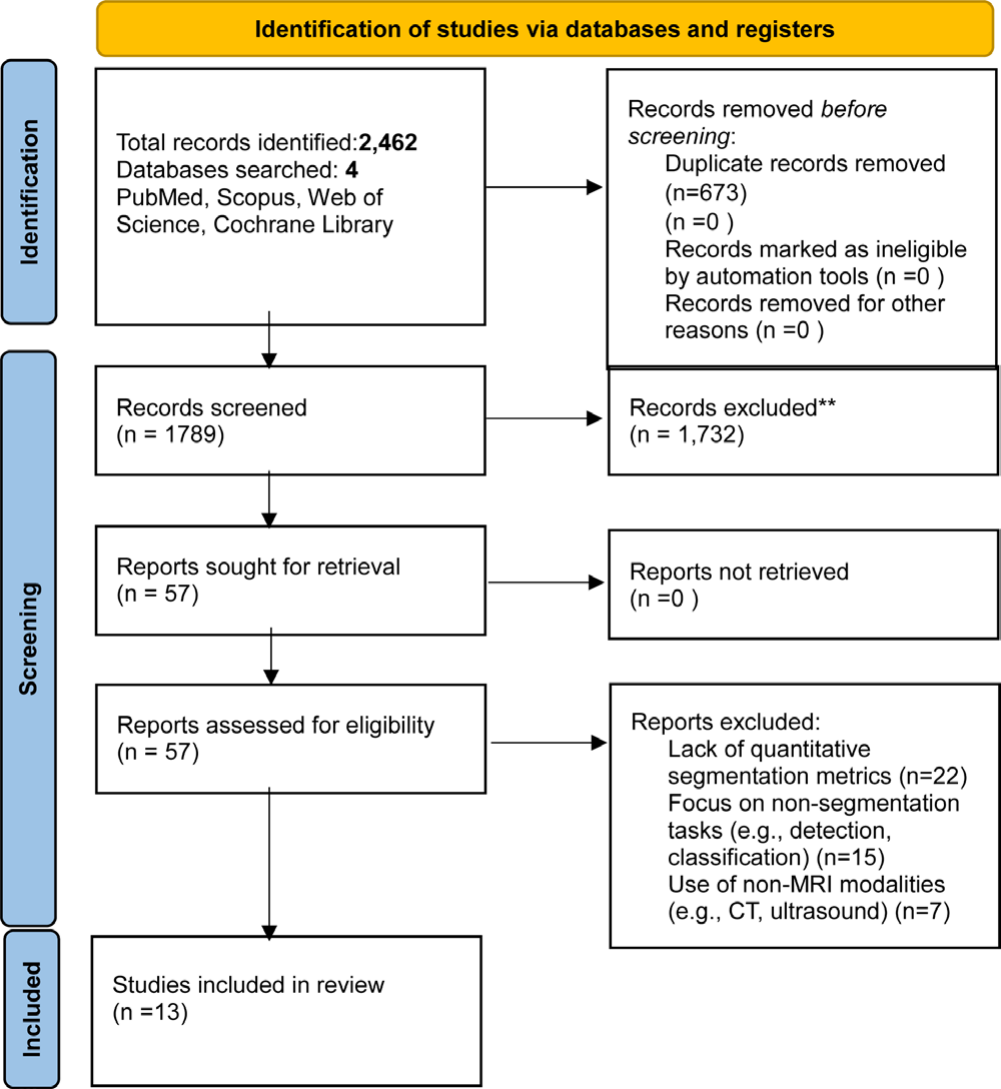
PRISMA flow diagram illustrating the study selection process for deep learning-based HCC segmentation in MRI. HCC = hepatocellular carcinoma, MRI = magnetic resonance imaging, PRISMA = preferred reporting items for systematic reviews and meta-analyses.

A systematic literature search across PubMed, Scopus, Web of Science, and Cochrane Library identified 2462 records relevant to DL for HCC segmentation in MRI in Table 3. After deduplication using EndNote X9, 1789 unique records remained. Two independent reviewers screened titles and abstracts, excluding 1732 records that did not meet eligibility criteria (e.g., non-DL methods, non-MRI modalities, or non-segmentation tasks). Full-text evaluation of 57 articles resulted in the inclusion of 13 studies meeting all criteria: utilization of DL models for HCC segmentation in MRI, reporting of quantitative performance metrics, involvement of human subjects with confirmed HCC, peer-reviewed original research, and English-language publication. The selection process is documented in a PRISMA flow diagram (Fig. 1). Reasons for exclusion at the full-text stage included lack of quantitative segmentation metrics (n = 22), focus on non-segmentation tasks (e.g., detection, classification; n = 15), and use of non-MRI modalities (e.g., CT, ultrasound; n = 7). See results summary in Tables 3–7.

**Table 3:** Number of records retrieved from each database.

Database	Records retrieved
PubMed	1012
Scopus	674
Web of science	456
Cochrane library	320
Total	2462

**Table 4:** Characteristics of included studies for deep learning-based HCC segmentation in MRI.

Study (Year)	Country	Sample size (HCC patients/lesions)	Study design	Data source	HCC focus	DL architecture	Key objective
Gatos et al^[12]^ (2017)	Greece	19 HCC lesions	Retrospective	Clinical MRI (Siemens 1.5T, 2013–2015)	HCC and other lesions (benign, metastases)	CNN-based CAD	Segment and classify liver lesions on T2-weighted MRI
Gross et al^[13]^ (2021)	USA	219 HCC patients	Retrospective	Institutional T1-weighted MRI (2008–2019)	HCC only	3D CNN	Assess 3D liver segmentation across BCLC stages
Hänsch et al^[14]^ (2022)	Germany	44 HCC patients	Retrospective	Internal LAVA DCE-MRI dataset	HCC only	Anisotropic 3D U-Net	Improve tumor segmentation in late-phase DCE-MRI
Liu et al^[15]^ (2021)	China	80 HCC patients	Randomized controlled trial	Hospital MRI scans (2018–2021)	HCC only	CNN	Guide doxorubicin nanopreparation release using CNN segmentation
Patel et al^[16]^ (2024)	USA	312 HCC patients	Retrospective	Six datasets (CHAOS, DLDS, AMOS, ATLAS, etc)	HCC only	nnUNet, PocketNet, Swin UNETR	Develop generalizable DL model for liver segmentation
Quinton et al^[17]^ (2024)	France	90 HCC patients	Comparative analysis with Bayesian optimization	ATLAS 3D CE-MRI dataset	HCC only	CNNs, transformers, hybrids	Optimize neural architectures for liver and tumor segmentation
Said et al^[18]^ (2023)	Thailand	292 HCC patients	Retrospective	MRI scans (08/2015–06/2019)	HCC only	CNN	Evaluate semiautomated HCC segmentation across sequences
Stollmayer et al^[19]^ (2025)	Germany	602 HCC patients	Retrospective	Gadoxetate-enhanced MRI (05/2005–09/2022)	HCC only	nnU-Net	Assess HCC risk and LI-RADS classification with nnU-Net
Wang et al^[10]^ (2023)	China	105 HCC patients	Retrospective	Sun Yat-Sen and TCIA MRI (2015–2020)	HCC only	UNet++	Validate UNet++ for liver and tumor segmentation
Ye et al^[20]^ (2024)	China	51 HCC patients	Retrospective	Gd-EOB-DTPA-enhanced MRI (2012–2015)	HCC only	DCNN, DFN	Develop semiautomatic HCC segmentation with radiologist aid
Luo et al^[21]^ (2024)	China	382 patients/ 466 lesions (internal); 51 patients/ 62 lesions (external)	Retrospective	DCE-MRI from 2 hospitals (2016–2021 internal; 2018–2021 external)	HCC detection and segmentation	Modified U-Net with multi-phasic input	Develop an automatic DL model for HCC detection and segmentation using DCE-MRI, achieving high accuracy and low false positives
Sui et al^[22]^ (2021)	China	100 HCC patients	Retrospective	MR k-space data (hospital dataset, 2016–2021)	Liver, renal, and HCC lesion segmentation; image reconstruction	Dual U-Net (multitask learning, RecSeg)	Develop a multitask learning model for simultaneous MRI reconstruction and segmentation of liver, renal organs, and HCC lesions, improving performance under accelerated imaging.
Gao et al^[23]^ (2024)	China	386 HCC patients	Retrospective	Single-center dataset	HCC only	Develop a multi-modality segmentation model to learn inter-phase correlations for accurate HCC segmentation	Transformer-based progressive attention segmentation framework (TPA) with multi-modality attention transformer module (MAT)

BCLC = Barcelona Clinic Liver Cancer, CAD = computer-aided diagnosis, CNN = convolutional neural network, DCE-MRI = dynamic contrast-enhanced magnetic resonance imaging, DCNN = deep convolutional neural network, DFN = deep fusion network, DL = deep learning, HCC = hepatocellular carcinoma, MRI = magnetic resonance imaging, LAVA = liver acquisition with volume acceleration.

**Table 5:** Summary of MRI sequences, DL architectures, performance metrics, and challenges in studies on HCC segmentation.

Study	MRI sequences	Field strength	DL architecture	Dataset size (slices/volumes)	Preprocessing techniques	Computational resources	Ground truth definition	Performance metrics	Validation method	Generalizability testing	Clinical application	Challenges
Gatos et al^[12]^ (2017)	Nonenhanced T2-weighted transverse	1.5T (Siemens MAGNETOM symphony)	Probabilistic neural network (PNN)	71 2D slices	EDMFCM segmentation, CWT edge detection, feature extraction (42 textural, 12 morphological), stepwise regression	Not reported*	Manual radiologist delineations, validated by ceMRI/biopsy	DSC: 0.91 ± 0.12; PNN accuracy: 90.1% (94.1% benign, 91.4% HCC, 94.1% metastasis); AUC: 0.88	Leave-one-out (LOO), ROC analysis	Not tested	Diagnostic aid for nonenhanced MRI, reduces invasive procedures	Small dataset, intensity overlap, manual ROI dependency, noise sensitivity, long processing time
Gross et al^[13]^ (2021)	Arterial-phase T1-weighted	1.16T, 1.5T, 3T	3D U-Net with residual units	Not reported* (3D volumes)	Isotropic voxel spacing (2 mm^3^), intensity scaling, random 3D patch extraction	NVIDIA RTX 2080 Ti GPU	Manual segmentations by medical student, supervised by radiologist	AS-Net: DSC 0.954 ± 0.018, MHD 3.500 ± 4.033 mm, MAD 0.750 ± 0.370 mm; EIS-Net: DSC 0.946 ± 0.032, MHD 5.812 ± 8.822 mm, MAD 1.243 ± 1.901 mm	70/15/15% train/validation/test split, Wilcoxon signed-rank test	Tested across all BCLC stages; AS-Net outperformed EIS-Net on advanced stages	Liver volumetry for treatment planning, preprocessing for cancer detection	Retrospective data, treatment-naïve HCC bias, skewed toward early BCLC stages, no multi-site validation
Hänsch et al^[14]^ (2022)	Late hepatocellular phase, LAVA DCE-MRI	Not reported*	Anisotropic 3D U-Net (aU-Net)	Not reported* (3D volumes)	Liver mask dilation, patch sampling	Not reported*	Manual segmentations by multiple raters	DSC: A1 0.732 ± 0.210, A2 0.689 ± 0.214; F1-score: 0.59	Train/validation/test split, Wilcoxon signed-rank test	Not explicitly tested	Liver tumor segmentation for therapy planning (e.g., SIRT)	Limited dataset size, no public MRI challenge data, poor performance for small lesions
Liu et al^[15]^ (2021)	T1-weighted (pre/post-contrast), T2-weighted	Not reported*	3D CNN (U-Net-based) with XGBoost post-processing	Not reported* (3D volumes)	Intensity normalization, resizing*	Not reported*	Manual segmentation by radiologists*	DSC: 0.78 ± 0.05; Accuracy: 80 ± 6.25%; Sensitivity: 0.82; Specificity: 0.79; AUC: 0.85	Train/test split (80/20%), ROC curve	Not explicitly tested	Guiding targeted doxorubicin therapy	Small sample size, single-source data, limited preprocessing details, no multicenter validation
Quinton et al^[17]^ (2024)	Contrast-enhanced MRI (CE-MRI)	Not reported*	Seven architectures (CNNs: nnUNet, SegmentationNet; Transformer: VT-UNet; Hybrids: nnFormer, Swin-UNETR, TransBTS, UNETR)	~4000–12,240 2D slices (44–136 slices/patient)	Bias field correction, patch size optimization, data augmentation	32 GB GPU, HPC resources (IDRIS, GENCI)	Manual delineation by experienced radiologist using MIM SurePlan LiverY90	DSC: 0.85–0.92; HD: 2.5–5.0 mm; Precision: 0.87–0.94; Recall: 0.83–0.90	Validation set (12 images), sliding window (50% overlap), exponential moving average of Generalised DSC	Limited to ATLAS dataset (single-center, University Hospital of Dijon)	Liver/tumor segmentation for SIRT dosimetry	Limited dataset diversity, long training times (up to 144h), sequential Bayesian search constraints
Said et al^[18]^ (2023)	T2WI, T1WI (pre/post-contrast: AP, PVP, DP, HBP), DWI, ADC	1.5T (212 patients), 3T (80 patients)	2.5D U-Net, 3D U-Net	Not reported* (3D volumes)	N4BiasFieldCorrection, DICOM to NIfTI conversion, zero-padding, resampling	2 NVIDIA 1080Ti GPUs, PyTorch	Manual segmentation by 3 radiologists using SPYD software	DSC: 0.80–0.87	Train (n = 195), val (n = 66), test (n = 31); DSC evaluation	Multi-vendor MRI systems (single-center)	Semiautomated HCC segmentation for diagnosis, treatment planning, radiomics	Single-center data, retrospective design, variable MRI protocols, lower DSC for small lesions and ADC/AP sequences
Stollmayer et al^[19]^ (2025)	NCE, AP, PVP, TRA, HBP, IP, OOP, T2H, T2B, T2LTE, DWI (low, medium, high b-values), ADC	1.5T, 3T	nnU-Net (2D, 3D full/low res, 3D cascade)	Not reported* (3D volumes)	DICOM to NIfTI conversion, co-registration to NCE, zero-value replacement for missing images	GPU-based (nnU-Net pipeline)*	Manual segmentation by radiology trainee and junior radiologist, proofread by expert radiologist, LI-RADS v2018 classification	DSC: 0.83–0.89; Sensitivity: 0.85; PPV: 0.82; F1: 0.84; CCC: 0.90; Cohen kappa: 0.78	Train (n = 383), internal test (n = 219), external test (n = 16); lesion detection, classification, segmentation evaluation	External validation on modified LiverHccSeg dataset; heterogeneous MRI scanners	Automated LI-RADS-based HCC risk mapping, tumor burden quantification, radiomics, clinical decision support	Single-expert ground truth, no separate LI-RADS feature evaluation, lower performance for LR-3/LR-4/LR-M lesions
Wang et al^[10]^ (2023)	Arterial phase, T2-weighted	Not reported* (GE Discovery MR 750)	UNet++ (2D and 3D)	Not reported* (3D volumes)	Intensity normalization, resizing, window-level adjustment, voxel size standardization, rigid registration, background removal	Not reported*	Manual segmentation by 2 radiologists (5 and 7 years’ experience) using 3D Slicer, consensus via discussion	DSC: 0.82–0.88 (liver), 0.61–0.69 (tumor)	Train (n = 83), val (n = 11), internal test (n = 11), external test (n = 9)	External validation on TCIA dataset (9 cases)	Automated liver/tumor segmentation, radiomics, improved delineation efficiency	High false positivity rate, limited public data, complex MRI protocols, small tumor size challenges
Ye et al^[20]^ (2024)	Hepatobiliary phase (HBP), portal venous phase (PVP)	3.0T	DCNN, DFN (U-Net-based, fully convolutional)	417 2D image pairs (HBP, PVP)	B-spline nonrigid registration, resampling to 256 × 256, data augmentation (rotation, scaling, contrast adjustment, noise, mirroring)	Workstation (E5-2650 v3 CPU, NVIDIA GeForce 1080TI GPU), Keras, TensorFlow	Manual contouring by radiologist (>10 yr experience), reviewed by independent radiologist (>10 yr experience)	DSC: 0.84 ± 0.04; Precision: 0.86; Recall: 0.83	Leave-one-out cross-validation (LOOCV)	Not performed (requires multi-center validation)	Semiautomatic HCC segmentation, faster/stable vs moderate-experience radiologist contouring	Semiautomated method reliant on radiologist intervention, 2D network limitations, small sample size, lack of multi-center validation
Luo et al^[21]^ (2024)	Dynamic contrast-enhanced (DCE) MRI (multi-phasic)	Not specified	Modified U-Net for multi-phasic input	volumes; 382 patients (466 lesions) internal, 51 patients (62 lesions) external	Phase registration, automatic liver region extraction, connected component analysis for post-processing	Not specified	Pathologically confirmed HCC lesions, manually contoured (details not specified)	Dice similarity coefficient (DSC), sensitivity, precision, false positives per patient	Five-fold cross-validation (training-validation set), independent test set, external validation	External validation on 51 patients from another hospital	Automated HCC detection and segmentation for improved clinical management	Limited prior research on DCE-MRI-based HCC segmentation, potential variability in multi-phasic imaging protocols
Sui et al^[22]^ (2021)	Not specified	Not specified	Dual U-Net (multitask learning, RecSeg)	Not specified	Not specified	Not specified	Manual contouring by an experienced radiologist	Dice score, PSNR, SSIM	Not specified	Not performed	Simultaneous MRI reconstruction and segmentation of liver, renal organs, and HCC lesions	Long imaging times, artifacts in accelerated MRI, challenges in automatic lesion segmentation for low-quality images
Gao et al^[23]^ (2024)	DCE-MRI (multi-phase)	Not specified	Two-stage progressive attention segmentation framework (TPA) with multi-modality attention transformer module (MAT)	386 cases (single-center) + 83 cases (multi-center); slices/volumes not specified	Not specified	Not specified	Radiologist annotations	Dice coefficient: 0.822 (internal), 0.772 (external), 0.829/0.791 (subgroup)	Train/validate/test split	External multi-center test (83 cases); subgroup analysis for weak inter-phase correlation	HCC segmentation for diagnosis and measurement	HCC heterogeneity, inconsistent lesion appearance across DCE-MRI phases

Notes: indicates data not reported in the original study; inferred or assumed where possible (e.g., GPU use for nnU-Net, manual segmentation for Liu et al).

ADC = apparent diffusion coefficient, AFPR = average false positivity rate, AP = arterial phase, BCLC = Barcelona Clinic Liver Cancer, CCC = concordance correlation coefficient, CE-MRI = contrast-enhanced MRI, DCE-MRI = dynamic contrast-enhanced MRI, DL = deep learning, DSC = dice similarity coefficient, DWI = diffusion-weighted imaging, FLLs = focal liver lesions, HBP = hepatobiliary phase, HD = Hausdorff distance, IoU = intersection over union, LAVA = liver acquisition with volume acceleration, LI-RADS = liver imaging reporting and data system, LOOCV = leave-one-out cross-validation, MAD = mean absolute distance, MHD = modified Hausdorff distance, PSNR = peak signal-to-noise ratio, PVP = portal venous phase, ROI = region of interest, SIRT = selective internal radiation therapy, T1WI/T2WI = T1/T2-weighted imaging. performance metrics prioritize DSC/IoU where reported; secondary metrics (e.g., accuracy, AUC) included if relevant. challenges are categorized as technical (e.g., dataset size, preprocessing), clinical (e.g., lesion heterogeneity), or imaging-related (e.g., protocol variability).

**Table 6:** Summary of DSC performance by model architecture.

Model type	Number of studies	Median DSC	DSC range
U-Net/ CNN variants	7	0.83	0.74–0.954
Transformer-based	2	0.79	0.772–0.829
Hybrid CNN-transformer	2	0.86	0.83–0.89

CNN = convolutional neural network, DSC = dice similarity coefficient.

**Table 7:** Clinical applications of DL models for HCC segmentation.

Study	Clinical application	Use case description
Gatos et al^[12]^ (2017)	Diagnosis	CAD tool to classify lesions and reduce biopsy need
Liu et al^[15]^ (2021)	Drug delivery	Guided doxorubicin nanopreparation via segmentation
Stollmayer et al^[19]^ (2025)	Risk stratification	Automated LI-RADS classification and HCC risk mapping
Ye et al^[20]^ (2024)	Diagnostic aid	Faster segmentation with radiologist intervention
Luo et al^[21]^ (2024)	Detection & planning	Automatic HCC detection in DCE-MRI for clinical decisions
Quinton et al^[17]^ (2024)	Dosimetry	Tumor segmentation for selective internal radiation therapy (SIRT) planning
Gross et al^[13]^ (2021)	Treatment monitoring	Liver segmentation across BCLC stages for longitudinal follow-up

BCLC = Barcelona Clinic Liver Cancer, CAD = computer-aided diagnosis, DCE-MRI = dynamic contrast-enhanced magnetic resonance imaging, DL = deep learning, HCC = hepatocellular carcinoma, LI-RADS = liver imaging reporting and data system.

Dataset sizes (e.g., number of slices or volumes) were not consistently reported across studies (e.g., Gross et al, 2021; Liu et al, 2021), potentially limiting the assessment of model training robustness (Table 2).

**Figure 2.**
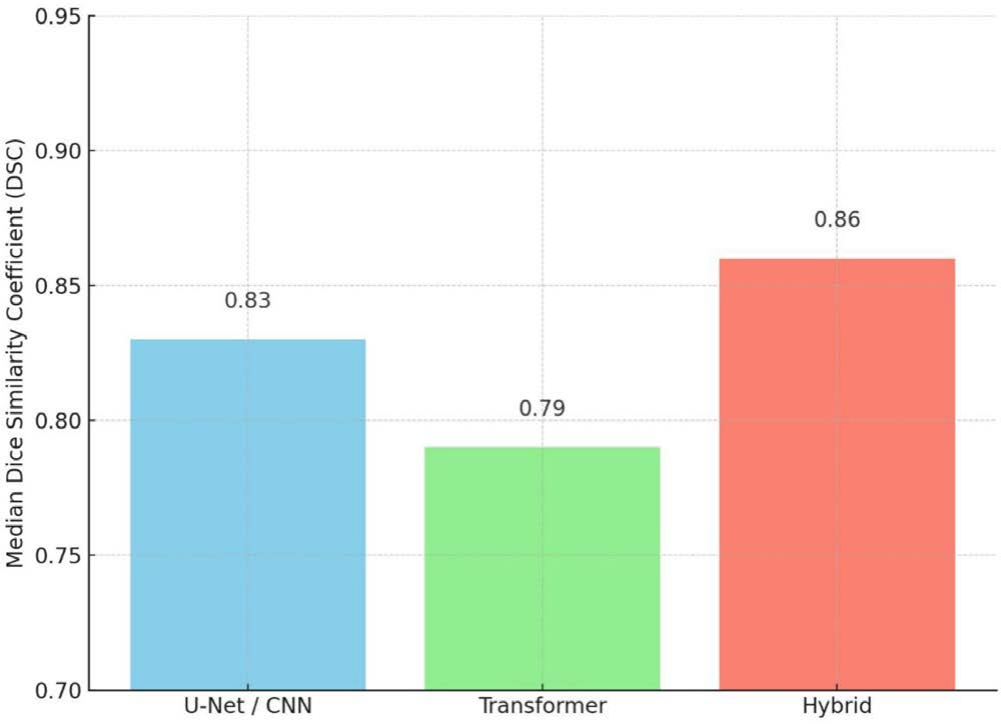
Median dice similarity coefficient (DSC) by model type.

To provide a consolidated view of segmentation performance, summary statistics of DSCs were computed across different model types. U-Net-based models (n = 7 studies) demonstrated a median DSC of 0.83 (range: 0.74–0.954), while transformer-based models (n = 2) achieved a median of 0.79 (range: 0.772–0.829). Hybrid architectures combining CNNs and transformers (n = 2) reported a median DSC of 0.86 (range: 0.83–0.89). These results are summarized in Table 6 and Figure 2.

While clinical applications were reported in several studies, these were not consistently mentioned. Table 7 categorizes each study by its primary application domain, highlighting how DL-based segmentation supports clinical workflows such as diagnosis, treatment planning, and risk assessment. This summary underscores the translational relevance of DL models in routine radiology and oncology practice.

### 3.2. Risk of bias assessment

**Figure 3.**
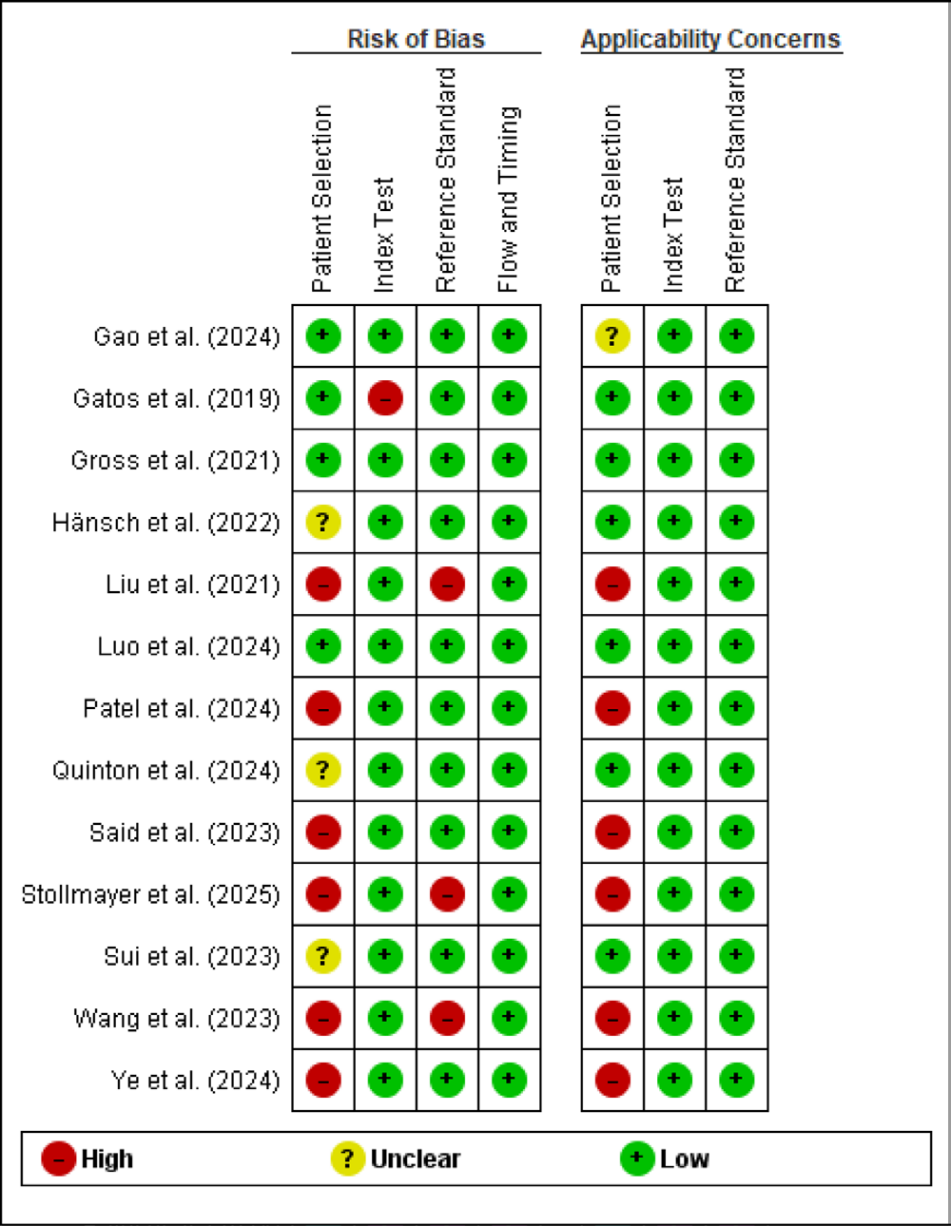
QUADAS-2 risk of bias and applicability concerns assessment for included studies. QUADAS-2 = quality assessment of diagnostic accuracy studies-2.

The methodological quality of the 13 included studies was evaluated using the QUADAS-2 tool, tailored for segmentation studies, to assess RoB and applicability concerns across 4 domains: patient selection, index test, reference standard, and flow and timing. Most studies exhibited low RoB in the index test (11/13) and flow and timing (13/13) domains, reflecting robust DL model methodologies with clear train/test splits and consistent MRI protocols. However, patient selection posed a high RoB in 8 studies (e.g., Hänsch et al, 2022; Patel et al, 2024) due to nonconsecutive enrollment or unclear inclusion criteria, potentially introducing selection bias and limiting generalizability. High applicability concerns in patient selection for 5 studies (e.g., Liu et al, 2021; Ye et al, 2024) arose from single-center datasets, which may limit relevance to diverse HCC populations with varying etiologies and imaging protocols. The reference standard domain showed high RoB in 3 studies (e.g., Liu et al, 2021; Stollmayer et al, 2025) due to insufficient details on segmentation expertise or inter-observer reliability, which could affect ground truth reliability. Annotation variability in manual ground truths, often stemming from inter-observer differences in delineating heterogeneous HCC lesions, likely influenced reported Dice scores by introducing inconsistencies in training data. For instance, in studies with unclear inter-observer reliability, such as Liu et al (2021) and Stollmayer et al (2025), this variability may have contributed to lower DSCs, including 0.78 ± 0.05 in Liu et al, as models trained on variable annotations tend to underperform on ambiguous boundaries. Conversely, studies with robust annotation protocols, such as multiple expert reviews in Gross et al (2021), achieved higher DSCs of 0.954 ± 0.018, highlighting the need for standardized ground truth generation to minimize bias and improve segmentation accuracy. Applicability concerns were generally low for index test and reference standard domains, as studies aligned with the review’s focus on HCC segmentation in MRI, but high applicability concerns in patient selection for 5 studies (e.g., Liu et al, 2021; Ye et al, 2024) arose from single-center datasets or mixed etiologies, reducing relevance to diverse HCC populations. These findings suggest that while technical aspects of DL-based segmentation are robust, improvements in patient selection strategies and reference standard reporting are critical to enhance study quality and clinical applicability (Fig. 3).

## 4. Qualitative synthesis and discussion

This systematic review synthesizes findings from 13 studies on DL applications for HCC segmentation in MRI, elucidating model architectures, performance metrics, and persistent challenges that shape their clinical potential and limitations. A comparative analysis reveals distinct patterns in segmentation efficacy, clinical utility, and barriers across technical, clinical, and imaging domains, offering insights into the state of the field and directions for future research.

Convolutional neural network (CNN)-based architectures, particularly U-Net and its variants, dominated the included studies due to their established efficacy in medical image segmentation. For instance, Gross et al (2021) utilized a 3D U-Net (AS-Net) across all Barcelona Clinic Liver Cancer stages, achieving a DSC of 0.954 ± 0.018, surpassing their early-intermediate-stage-net (EIS-Net) with a DSC of 0.946 ± 0.032 (*P* < .001).^[13]^ Similarly, Wang et al (2023) employed UNet++ to attain robust liver segmentation (DSC 0.82–0.88) but faced challenges with tumor segmentation (DSC 0.61–0.69), particularly for smaller or less distinct lesions.^[10]^ Hänsch et al (2022) and Liu et al (2021) advanced U-Net applications through anisotropic 3D architectures and multi-model training, yielding DSCs of 0.74 and 0.78 ± 0.05, respectively.^[14,15]^ However, both studies noted limitations in detecting small lesions, with Hänsch et al reporting an F1-score of 0.59 compared to 0.76 for human raters.

Emerging architectures, including transformers and hybrid models, demonstrated adaptability to complex HCC datasets. For example, a 2024 study evaluated 7 architectures (CNNs, transformers, and hybrids) on the ATLAS dataset, achieving DSCs of 92.3 to 95.1% for liver segmentation and 41.5 to 69.7% for tumors, with Bayesian hyperparameter tuning enhancing performance by 1.7% for liver and 5.0% for tumors. Gao et al (2024) introduced a two-stage progressive attention segmentation model, leveraging inter-phase correlations in dynamic contrast-enhanced (DCE)-MRI to achieve DSCs of 0.772 to 0.829, outperforming state-of-the-art models. Similarly, Sui et al (2023) developed a multitask learning model (RecSeg) that excelled in simultaneous MRI reconstruction and segmentation (DSC 0.86, peak signal-to-noise ratio 32.39), underscoring the potential of integrated approaches in accelerated imaging scenarios.^[22,23]^ While transformer and hybrid models offer performance gains, such as 1.7 to 5.0% DSC improvements in a 2024 study through Bayesian tuning, their higher computational costs – such as extended training times up to 144 hours for nnFormer – may limit routine clinical practice unless offset by efficiency in deployment or hardware optimizations. Clinical applications of DL-based HCC segmentation spanned diagnostic support, treatment planning, and risk assessment. Gatos et al (2017) developed a computer-aided diagnosis system for nonenhanced T2-weighted MRI, achieving a mean segmentation overlap of 0.91 ± 0.12 and classification accuracies of 90.1 to 94.1% for distinguishing HCC from benign and metastatic lesions, potentially reducing the need for invasive procedures. Stollmayer et al (2025) applied nnU-Net for HCC risk assessment using liver imaging reporting and data system v2018, reporting high sensitivity (0.83–0.90) for LR-5 lesions but reduced accuracy for LR-3, LR-4, and LR-M lesions, reflecting challenges with lesion heterogeneity.^[19]^ Ye et al (2024) integrated deep CNNs and deep fusion networks with radiologist intervention, achieving a DSC of 0.83 and reducing segmentation time from 15 to 11 minutes, highlighting potential for enhanced clinical efficiency.^[20]^ Luo et al (2024) reported a modified U-Net with DSCs of 0.75 to 0.81 and low false positives (0.12–0.14 per patient), emphasizing its utility in automated HCC detection and segmentation.^[21]^ DL models complement radiologist-driven workflows by facilitating liver imaging reporting and data system reporting through automated risk mapping, as demonstrated in Stollmayer et al (2025), and supporting treatment planning via precise lesion delineation for radiotherapy or surgery, as shown in Gross et al (2021). Existing studies demonstrate measurable benefits, such as Ye et al (2024) reporting a reduction in segmentation time from 15 to 11 minutes with radiologist intervention, and Gatos et al (2017) achieving classification accuracies of 90.1 to 94.1%, potentially improving diagnostic accuracy over manual methods alone by reducing inter-observer variability and enabling faster decision-making in oncology practice.

Performance variability was closely tied to MRI sequence types and tumor characteristics. Said and colleagues in 2023 found that single-slice segmentation (DSC 0.442–0.778) outperformed volumetric approaches (DSC 0.305–0.667), with high b-value diffusion-weighted imaging yielding superior results, indicating sequence-specific optimization challenges.^[18]^ Patel et al (2024) demonstrated generalizability across 6 datasets using nnUNet and PocketNet (DSCs > 0.9), though Swin UNETR underperformed, suggesting architecture-specific limitations.^[16]^ Challenges were categorized into technical (e.g., overfitting due to limited dataset sizes), clinical (e.g., lesion heterogeneity impacting accuracy), and imaging-related (e.g., MRI protocol variability) domains to ensure clarity and avoid overlap.

Limited dataset sizes consistently hindered model training and generalizability. Gatos et al (2017) noted that a small dataset of 19 HCC lesions led to potential overfitting, compounded by intensity overlap issues and manual region-of-interest dependency.^[12]^ Ye et al (2024) and Liu et al (2021) highlighted small sample sizes (51 and 80 patients, respectively) as barriers to robust model development, with the former noting limitations of 2D network architectures.^[15]^ A 2024 study reported computational constraints, with training times reaching 144 hours for transformer-based models like nnFormer on a dataset of 60 images. Wang and colleagues in 2023 observed high false positivity rates (1–1.6 per patient) and limited public data availability, while Hänsch et al (2022) struggled with small lesion detection due to insufficient training data. Conversely, studies with larger datasets, such as Stollmayer et al (2025) (602 patients) and Patel et al (2024) (819 patients), still faced generalizability challenges due to single-center designs, indicating that dataset size alone is insufficient without diversity.

Lesion heterogeneity posed a significant clinical challenge, particularly for tumors with variable contrast uptake or small sizes. Hänsch et al (2022) and Said et al (2023) reported lower DSCs for small lesions (e.g., DSC 0.305–0.667 for volumetric segmentation in the latter), with Said and colleagues noting specific difficulties in arterial phase and apparent diffusion coefficient sequences. Stollmayer et al (2025) highlighted reduced accuracy for LR-3, LR-4, and LR-M lesions, reflecting difficulties in capturing diverse HCC phenotypes. Gao and colleagues in 2024 addressed heterogeneity by leveraging inter-phase correlations in dynamic contrast-enhanced magnetic resonance imaging (DCE-MRI), yet noted inconsistent lesion appearance across phases. Liu et al (2021) and Ye et al (2024) emphasized the necessity of radiologist intervention, with the latter’s deep fusion networks-R approach improving DSC to 0.83 through manual refinement, underscoring the current reliance on human expertise to address clinical variability.^[19,20]^

Imaging-related challenges, including MRI protocol variability and artifacts, further impacted model performance and generalizability. Said and colleagues in 2023 noted inconsistent segmentation performance across sequences (e.g., T1-weighted imaging, diffusion-weighted imaging, apparent diffusion coefficient) due to protocol variability, particularly for volumetric approaches. Luo et al (2024) and Gao et al (2024) highlighted challenges with multi-phasic DCE-MRI protocols, with the former noting limited prior research on DCE-MRI-based HCC segmentation. Sui and colleagues in 2023 faced artifacts in accelerated MRI, affecting segmentation in low-quality images despite achieving a high DSC of 0.86. Gross et al (2021) reported a bias toward early Barcelona Clinic Liver Cancer stages in retrospective data, limiting applicability across diverse imaging vendors, while Wang et al (2023) struggled with complex MRI protocols, exacerbating false positivity rates for small tumors.^[13]^

This comparative analysis reveals a spectrum of challenges and mitigation strategies. Studies with smaller datasets (e.g., Gatos et al, 2017; Ye et al, 2024) were primarily constrained by technical limitations, such as overfitting and manual dependency, while larger studies (e.g., Stollmayer et al, 2025; Patel et al, 2024) emphasized generalizability and clinical integration challenges, including the lack of external validation across diverse populations. Imaging-related challenges were more pronounced in studies using complex sequences like DCE-MRI (e.g., Luo et al, 2024; Gao et al, 2024), where phase variability introduced complexity, compared to single-sequence studies like Gatos et al (2017) on T2-weighted MRI, which faced simpler issues like noise sensitivity. Lesion heterogeneity remained a universal clinical challenge, with studies incorporating radiologist input (e.g., Ye et al, 2024; Liu et al, 2021) or advanced architectures (e.g., Gao et al, 2024) demonstrating improved mitigation, suggesting synergy between human expertise and innovative model design.^[12]^

To address these challenges, a multi-faceted approach is essential. Technical limitations, such as small datasets, could be mitigated through the development of public, multi-center MRI datasets, as emphasized by Wang et al (2023) and Patel et al (2024). Clinical challenges, particularly lesion heterogeneity, necessitate hybrid approaches integrating DL with radiologist oversight, as demonstrated by Ye et al (2024), or advanced architectures like transformers that capture long-range dependencies. Imaging-related challenges require standardized MRI protocols, particularly for complex sequences like DCE-MRI, to enhance model robustness across vendors and centers. Collectively, these findings underscore the transformative potential of DL for HCC segmentation in MRI while highlighting the critical need for collaborative dataset development, standardized imaging protocols, and integrated clinical workflows to translate research advancements into routine practice. Future research should prioritize: the creation of multi-institutional, publicly available MRI datasets with standardized imaging protocols; evaluation frameworks using both internal and external validation with diverse etiologies; and hybrid training paradigms incorporating radiologist feedback via active learning or human-in-the-loop methods to improve model reliability in challenging cases.

## 5. Conclusion

This systematic review highlights the transformative potential of DL for HCC segmentation in MRI, with U-Net-based and transformer architectures achieving DSCs of 0.61 to 0.954 across diverse clinical applications. Despite robust technical performance, challenges such as limited dataset sizes, lesion heterogeneity, and MRI protocol variability underscore the need for larger, multi-center datasets, standardized imaging protocols, and hybrid approaches integrating radiologist expertise to enhance generalizability and clinical utility. However, high RoB, small sample sizes, and single-center datasets limit the generalizability of current findings. To bridge the gap between research and clinical practice, future efforts should promote collaborative dataset development and explore hybrid AI-radiologist systems that balance automation with expert oversight.

## Acknowledgments

The authors wish to thank the reviewers and editors for their valuable feedback and contributions to improving this manuscript.

## Author contributions

**Conceptualization:** Mohammadreza Elhaie, Abolfazl Koozari.

**Data curation:** Abolfazl Koozari.

**Formal analysis:** Mohammadreza Elhaie, Abolfazl Koozari, Maryam Arjmandi.

**Investigation:** Mohammadreza Elhaie, Maryam Arjmandi.

**Methodology:** Maryam Arjmandi.

**Project administration:** Mohammadreza Elhaie, Nadia Najafizde.

**Supervision:** Mohammadreza Elhaie, Nadia Najafizde.

**Validation:** Mohammadreza Elhaie, Nadia Najafizde.

**Visualization:** Mohammadreza Elhaie.

**Writing – original draft:** Mohammadreza Elhaie.

**Writing – review & editing:** Mohammadreza Elhaie.
